# Response hierarchy models and their application in health and medicine: understanding the hierarchy of effects

**DOI:** 10.1186/s12913-020-05605-8

**Published:** 2020-09-15

**Authors:** James K. Elrod, John L. Fortenberry

**Affiliations:** 1 Willis-Knighton Health System, 2600 Greenwood Road, Shreveport, LA 71103 USA; 2 LSU Shreveport, 1 University Place, Shreveport, LA 71115 USA

**Keywords:** Marketing communications, Response hierarchy models, Hierarchy of effects models, Hospitals, Healthcare

## Abstract

**Background:**

Successful patient engagement pursuits naturally require healthcare providers to possess a detailed understanding of their target audiences, with one of the most important processes to comprehend being the manner in which they learn about particular establishments and decide to extend their patronage. While health services patronage pathways vary between and among consumers, general patronage patterns exist which can provide enlightenment regarding this important process. Achieving knowledge on this front can help healthcare providers maximize opportunities to engage audiences and acquire all-important market share.

**Discussion:**

The discipline of marketing, in part, focuses on customer engagement practices and, in describing the patronage process, it often uses what are referred to as response hierarchy models. Also known as hierarchy of effects models, these representations can help healthcare providers to understand the course through which individuals become customers of given establishments, aiding them particularly in devising appeals that can accelerate the patronage process. This particular article describes response hierarchy models, presents examples, and discusses the benefits that they offer healthcare institutions in their efforts to engage patients.

**Conclusions:**

As institutional viability and vitality are predicated on abilities to successfully attract and retain patients, healthcare establishments must direct keen attention toward developing associated skills. This necessitates that health and medical providers possess a detailed understanding of their target audiences, notably including the stages through which they pass on their way to becoming patrons. Response hierarchy models present the patronage process, depicting given stages, permitting insights which can assist healthcare providers in their quests to hasten desired exchange and capture market share.

## Background

Patients are vital for health and medical establishments, as without their patronage, institutional operations would not be possible [1–3]. As such, healthcare providers must work intensively over the course of organizational life to attract and retain customers, engaging them in a manner to entice their patronage and, ideally, capture their enduring loyalty [2, 4–7]. Successful patient engagement pursuits naturally require healthcare providers to possess a detailed understanding of their target audiences, with one of the most important processes to comprehend being the manner in which they learn about particular establishments and decide to extend their patronage [8, 9]. While health services patronage pathways vary between and among consumers—especially in terms of speed, given that some healthcare decisions must be made immediately, as in cases of emergency medical treatment—general patronage patterns exist which can provide enlightenment regarding this important process. Achieving knowledge on this front can help healthcare providers maximize opportunities to engage audiences and acquire all-important market share [2, 10, 11].

In seeking guidance related to patronage and its acquisition, one need not look further than the discipline of marketing which, in part, focuses on customer engagement practices, including market share development [12, 13]. In describing the patronage process, marketing academicians and practitioners often turn to what are referred to as response hierarchy models. Also known as hierarchy of effects models, these representations can help healthcare providers to understand the general course through which individuals become customers of given establishments [2, 11, 12, 14]. The stages traversed by consumers on their way toward becoming customers and patients indeed must be at the forefront of thought as healthcare institutions design their marketing communications campaigns, as any opportunity to craft conveyances in a manner that accelerates patronage affords obvious benefits for given health and medical establishments and those they serve [2]. This particular article describes response hierarchy models, presents examples, and discusses the benefits that they offer healthcare institutions in their efforts to engage current and prospective audiences.

## Definition, overview, and examples

Formally defined, a response hierarchy model is a depiction characterizing the patronage process; that is, the steps through which consumers pass on their way to becoming customers of given establishments. Known also as hierarchy of effects models, response hierarchy models are general representations depicting the stages leading to patronage [2, 11, 12, 14]. Understanding the patronage process is particularly helpful for crafting marketing communications in a manner to expedite the conversion of prospects into customers. With such knowledge, marketing communications campaigns can be structured to address prospects at various decision-making stages, hastening customer acquisition and market share development [2, 12, 14]. Response hierarchy models, while describing very complex processes, do so in a very simplistic, logical fashion, permitting most anyone to acquire a solid foundation of knowledge concerning the patronage process [2, 12]. Multiple versions of response hierarchy models exist, with each reflecting the particular ideas and beliefs of given authors as to how patronage processes work [12, 15, 16]. Of these models, AIDA and DAGMAR are among the most popular.

AIDA is one of the earliest response hierarchy models and it includes four stages: attention, interest, desire, and action [16–18]. First published by Strong in 1925 [18] and attributed to a nineteenth century work by Elmo Lewis [16], the AIDA model asserts that the attention of sought audiences must first be triggered, perhaps via advertising, direct marketing, or some other form of promotion, evoking interest on the part of targeted groups. Derived interest, in turn, compels prospective customers to research the given offering, forwarding inquiries or using other methods to investigate the particular item. If discovered to be capable of meeting or exceeding associated wants and needs, desire manifests, ultimately leading to action, whereby target audiences decide to extend their patronage, becoming customers of given establishments. Despite its age, AIDA remains a central tenet of many marketing publications [19].

Derived from the title of the book profiling the given model, *Defining Advertising Goals for Measured Advertising Results*, the DAGMAR Marketing Communications Spectrum includes five stages: unawareness, awareness, comprehension, conviction, and action [2, 11, 20, 21]. DAGMAR is very similar to AIDA, with the notable exception that it adds an unawareness stage at the beginning of the process. Although the unawareness stage could logically be assumed to precede AIDA’s attention stage, its inclusion in the DAGMAR model represents a descriptive improvement over AIDA. A further contribution of the DAGMAR model is that Colley [20] and Dutka [21], in presenting this work, directed attention toward marketing forces and countervailing forces and their impact on consumers across the spectrum, providing an important reminder of environmental influences. They additionally emphasized designing communications specific to given stages in order to hasten patronage decisions [2, 16, 20, 21].

While other versions of response hierarchy models exist, the AIDA and DAGMAR models provide a general sense of what these particular representations are seeking to illustrate. Importantly, it must be understood that these are general depictions that show pathways leading to patronage. Quite obviously, not everyone who gains an awareness of an offering advances to subsequent stages [2, 12], something that is especially the case for complex products, such as those provided by health services organizations.

Prospects newly aware of a particular medical procedure might, for example, not be suitable candidates for the given service, they might not have insurance or other means to pay for the procedure, they might not be able to overcome concerns regarding potential complications, and so on. These and related obstructions and reservations quite obviously will end their advancement along the patronage pathway. Other prospects, however, will find that they have the desire and means for the noted procedure and, as such, they will advance through associated stages to become customers and patients of the given healthcare establishment. And, of course, there are many scenarios where health services patronage occurs with little to no forethought, as in cases where life-threatening situations are encountered requiring immediate emergency treatment, resulting in instant adoption without consumers traversing through prior stages of response hierarchy models [2].

## Operational reflections

Beyond the value of simply envisioning the stages leading to patronage, adding a degree of insight into consumer behavior, response hierarchy models offer operational value. As indicated earlier, they can be used to help craft marketing communications, tailored to prospects at different stages of the patronage process [2, 16, 20, 21]. If evidence indicates that consumers in the marketplace do not possess foundational knowledge of a given healthcare entity, perhaps due to its recent introduction or a merger that sees it carry a new brand name, advertisements and related marketing communications can emphasize awareness building, helping audiences to acquire an understanding of the given healthcare provider. Assume, for example, that Evergreen Hospital and Meadowbrook Clinic merge, forming Emerald Hills Medical Center. As the two entities transition into one, operating under a new identity, customers in the marketplace will need to be informed of this, calling for an appeal introducing the new establishment, discussing its origins, and perhaps conveying benefits that can be expected from the union.

But suppose the particular healthcare establishment learns that foundational knowledge exists, however, prospects are not sufficiently engaged to investigate its service offerings. In such cases, marketing communications can be designed to emphasize education, giving prospects opportunities to learn about offerings and their potential benefits. Here, Emerald Hills Medical Center, realizing that the public is already well informed of its new identity resulting from the merger, might decide to emphasize in its marketing communications several key healthcare services provided by the institution, stimulating interest and potentially evoking desirability for the services in the hearts and minds of prospects.

If evidence indicates broad awareness and understanding of the establishment and its services, incentives can be used to compel prospects to extend their patronage and become customers of the given healthcare provider. Here, if Emerald Hills Medical Center sensed that prospects were nearing action, occupying advanced interest and desire stages, it might decide to, say, issue discount coupons for one or more of its services, motivating individuals to take action and become patrons.

Of course, when tailoring marketing communications to individuals occupying specific stages along patronage pathways, healthcare providers must be reasonably sure that the targeted consumers indeed occupy the particular stage or stages envisioned. Sometimes this is obvious, as in cases where healthcare establishments are new to the market and the public broadly does not possess knowledge of them, warranting awareness-building efforts. But other situations are more ambiguous, requiring the formulation of marketing communications in a manner to address consumers across the spectrum of decision making. Given the broad applicability and appeal of services typically offered by health and medical providers, scenarios requiring institutions to direct attention simultaneously toward prospects occupying different decision-making stages are rather common [1, 2, 8]. This is illustrated by the following example profiling Willis-Knighton Health System’s marketing communications approach, informed by response hierarchy models.

As a comprehensive provider of health services and holder of market leadership in the Ark-La-Tex region of the United States, Willis-Knighton Health System serves virtually every segment of the area’s population, characterized by residents who have lived in the region for enduring periods of time, complemented by a continuous flow of newcomers, hastened notably by the presence of prominent educational and military establishments. As such, the institution must ensure that marketing communications address individuals across the full range of stages of the patronage process. To facilitate comprehensive appeal, Willis-Knighton Health System’s marketing communications campaigns are carefully crafted to incorporate information relevant to consumers spanning the hierarchy of effects. Marketing communications, regardless of service line featured, typically incorporate foundational information about the system, along with avenues for gaining additional details (e.g., via telephone numbers, web links, etc.), supplying helpful content benefiting audiences at any decision point. Occasionally, such communications will incorporate deals (e.g., specials offering discounted services or free consultations), providing incentives particularly for those occupying advanced stages along the patronage pathway, typically also referencing foundational information for those occupying less advanced stages. When carefully formulated with response hierarchy models in mind, such campaigns address individuals most anywhere on the patronage spectrum, improving awareness, closing knowledge gaps, and enticing desire, helping advance patronage decisions with the ultimate goal being market share development.

As illustrated by Willis-Knighton Health System’s approach, response hierarchy models are very helpful for guiding marketing communications planning, ensuring that healthcare providers remain mindful that consumers vary in their developmental stages of patronage, necessitating conveyances suited to their respective places on decision-making spectrums. Response hierarchy models also usefully compel healthcare providers to acquire an enhanced understanding of the market dynamics enveloping their given institutions, as these details are most helpful for ascertaining the particular decision-making stage or series of stages on which to focus attention [2, 10]. Additional operational benefits are afforded by engaging in role playing exercises [22], whereby healthcare providers place themselves in the position of consumers at given stages of patronage and devise campaign elements anticipated to appeal to each stage, assisting health and medical establishments in fielding marketing communications campaigns inclusive to all audiences, helping to move them toward exchange and newfound status as customers and patients of given healthcare providers.

Crucially, once healthcare providers capture the patronage of prospects, converting them into customers, they certainly should not become complacent. Instead, they must remain highly attentive to customers after their initial patient experiences have concluded, addressing any resulting wants and needs, extending what effectively is post-purchase or post-adoption support. This will help bolster retention, a stage typically omitted from response hierarchy models, but vital for engendering loyalty and growing market share, prompting efforts by some researchers to revise these classic representations, accordingly [23–25]. Achieving success on both patient acquisition and patient retention fronts will yield burgeoning market share and all of the benefits afforded by such.

## Conclusions

As institutional viability and vitality are predicated on abilities to successfully attract and retain patients, healthcare establishments must direct keen attention toward developing associated skills. This necessitates that health and medical providers possess a detailed understanding of their target audiences, notably including the stages through which they pass on their way to becoming patrons. Response hierarchy models present the patronage process, depicting given stages, permitting insights which can assist healthcare providers in their quests to hasten desired exchange and capture market share. They are particularly helpful for crafting marketing communications in a manner to expedite the conversion of prospects into customers, warranting acquisition of an associated understanding, allowing health and medical organizations to more effectively engage patient populations.

## Data Availability

Not applicable.
